# Assessing the feasibility of harm reduction services for MSM: the late night breakfast buffet study

**DOI:** 10.1186/1477-7517-3-29

**Published:** 2006-10-03

**Authors:** Valerie J Rose, H Fisher Raymond, Timothy A Kellogg, Willi McFarland

**Affiliations:** 1 San Francisco Department of Public Health, AIDS Office, 25 Van Ness Avenue, Suite 500, San Francisco, CA 94102, USA; 2 Public Health Foundation Enterprises, Inc. (PHFE), Policy and Evaluation Research, PO Box 8528, Emeryville, CA 94662, USA

## Abstract

**Background:**

Despite the leveling off in new HIV infections among men who have sex with men (MSM) in San Francisco, new evidence suggests that many recent HIV infections are linked with the use of Methamphetamine (MA). Among anonymous HIV testers in San Francisco, HIV incidence among MA users was 6.3% compared to 2.1% among non-MA users. Of particular concern for prevention programs are frequent users and HIV positive men who use MA. These MSM pose a particular challenge to HIV prevention efforts due to the need to reach them during very late night hours.

**Methods:**

The purpose of the Late Night Breakfast Buffet (LNBB) was to determine the feasibility and uptake of harm reduction services by a late night population of MSM. The "buffet" of services included: needle exchange, harm reduction information, oral HIV testing, and urine based sexually transmitted infection (STI) testing accompanied by counseling and consent procedures. The study had two components: harm reduction outreach and a behavioral survey. For 4 months during 2004, we provided van-based harm reduction services in three neighborhoods in San Francisco from 1 – 5 a.m. for anyone out late at night. We also administered a behavioral risk and service utilization survey among MSM.

**Results:**

We exchanged 2000 needles in 233 needle exchange visits, distributed 4500 condoms/lubricants and provided 21 HIV tests and 12 STI tests. Fifty-five MSM enrolled in the study component. The study population of MSM was characterized by low levels of income and education whose ages ranged from 18 – 55. Seventy-eight percent used MA in the last 3 months; almost 25% used MA every day in the same time frame. Of the 65% who ever injected, 97% injected MA and 13% injected it several times a day. MA and alcohol were strong influences in the majority of unprotected sexual encounters among both HIV negative and HIV positive MSM.

**Conclusion:**

We reached a disenfranchised population of MA-using MSM who are at risk for acquiring or transmitting HIV infection through multiple high risk behaviors, and we established the feasibility and acceptability of late night harm reduction for MSM and MSM who inject drugs.

## Background

Following the initial spread of HIV among men who have sex with men (MSM) at the outset of the epidemic 25 years ago, estimates of new HIV infections among MSM in San Francisco decreased dramatically between 1988 and 1996 from as high as 8% per year in the mid 1980s to as low as 1% per year by 1996 [1]. From 1996 to 2001, HIV incidence rose again reaching about 2.2% per year [WMcF personal communication]. Since 2001, transmission appears to have leveled off at approximately 1.5% to 2.0% per year [2].

Despite the leveling off in new HIV infections across MSM as a whole, new evidence suggests that many recent HIV infections are linked with the use of Methamphetamine (MA). For example, among anonymous HIV testers in San Francisco, HIV incidence among MA users was 6.3% compared to 2.1% among non-MA users [3]. Recent research indicates that sexual behaviors known to increase risk for HIV transmission, such as unprotected anal intercourse, frequent and prolonged sexual activity and multiple sex partners are associated with MA use [4-24]. Of special concern are frequent users of MA and HIV positive men who use MA [25-27]. MA is a highly potent stimulant and can lead to frequent use, dependency and addiction; upon withdrawal, MA can cause severe psychological and physical symptoms [28,29]. Injecting MA creates increased risk for HIV transmission from both sexual and needle sharing behaviors among MSM and their partners [30-34].

Based on a population based behavioral surveillance study conducted by the San Francisco Department of Public Health (SFDPH), the prevalence of MA use among all MSM in San Francisco is estimated at 22% (HFR, personal communication). Among HIV negative MSM, 5% reported weekly use of MA and 9% of HIV positive men used MA weekly [35].

MSM who use MA pose a particular challenge to HIV prevention efforts due to the difficulty in reaching this group of MSM who are often active during very late night hours [HFR, personal communication, [36]]. The "Party and Play" study conducted by the SFDPH sought to assess this population during 2001–2002 by recruiting study participants between midnight and 4 a.m. in San Francisco parks and streets, near bars and cafes, adult bookstores and other popular cruising hangouts. The study found high HIV prevalence (31%) and extremely high levels of recent unprotected receptive (63%) and insertive anal sex (64%). In addition, an equivalent proportion of both HIV positive and HIV negative individuals in this population reported unprotected receptive (32%) and insertive anal sex (31%) with partners whose HIV serostatus was unknown or sero-discordant. The study population also reported high levels of injection (35%) and non-injection drug use (84%) [36].

The SFDPH Late Night Breakfast Buffet (LNBB) reported here significantly enhanced the concept and approach of the "Party and Play" study by testing the feasibility of providing harm reduction services, including needle exchange, using a mobile van; extending the hours of outreach to 5 a.m. and following up with MSM three months later to determine prevention and other services utilization.

The goal of the LNBB was to engage MSM who were not being reached through conventionally scheduled HIV prevention programs including needle exchange programs, and to reach MSM who may not find HIV prevention interventions geared towards non-injection drug users appropriate for their needs [27]. We chose a mobile intervention based on the success of similar studies/projects initiated by the SFDPH and literature demonstrating the effectiveness of delivering services to hard to reach populations via mobile vans [37-40]. This paper describes the results of the process evaluation of field based activities as well as the baseline results from study participants. Three month follow up and referral outcomes are reported in a separate paper.

## Methods

### Study Overview

The LNBB conducted fixed-site outreach using a 19-foot van to assess the acceptability and uptake of harm reduction services by a late night population. Clients were welcomed to the van by free access to water and nutritional snacks. The "buffet" of harm reduction services included: needle exchange, harm reduction information, oral HIV testing, urine based testing for gonorrhea and Chlamydia accompanied by brief client centered counseling and consent procedures. No incentives were provided for returning for HIV/STI test results; results and post test counseling were offered 7 days a week between 8 a.m. – 9 p.m. at the centrally located offices of the SFDPH. Specimens were analyzed at the SFDPH Public Health Laboratory using standard testing procedures.

### Study Subjects and Recruitment

Between July and October 2004, the van was parked in consistent locations in three neighborhood areas in San Francisco, three nights (i.e., Friday-Saturday-Sunday) per week from 1 – 5 a.m. These neighborhoods were: the Castro, a predominantly gay neighborhood, the South of Market, notable for drug dealing and drug use, and the Polk, where an established needle exchange program operates during the week until 9 p.m. Both the locations and times were determined from data collected in previous late night research conducted by the SFDPH [HFR personal communication, [36]] and data collected during a formative research phase which suggested that MSM and others were out late at night in particular parks, streets, cruising areas, alleys, near adult bookstores and sex clubs. Formative research included discussions with service providers and a focus group with substance users in a local drug treatment program.

Two staff members rotated the activities of needle exchange and HIV/STI screening and counseling at each site each week. The staff who conducted needle exchange or HIV/STI screening did so exclusively on any given night. Two additional staff greeted potential clients and conducted interviews. The majority of LNBB outreach was conducted by the same three staff members and the principal investigator.

In addition to the feasibility and acceptability aspects of the study, we also conducted a pilot behavioral risk and service utilization survey among MSM. The survey component was not linked to the feasibility aspect of the study (i.e., an MSM was not required to access services in order to be screened for the survey). Conversely, a male accessing services was asked if he would like to be screened to participate in the survey.

Consecutive, convenience sampling (i.e., each man who walked by and was willing to engage with staff) for the survey component was used to screen males. Screening consisted of an oral questionnaire to determine eligibility (e.g., male; self-reported to have had sex with men in the last 3 months, 18 years of age or older). Once eligibility was confirmed, potential participants were asked whether they were willing to provide locating information and to return for a follow up assessment in three months. Only those eligible men who agreed to provide locating information and could return in 3 months were enrolled in the study. An extensive "locator form" was used to enhance the potential of finding MSM for the follow up assessment. The form contained items such as telephone or pager numbers, addresses including e-mail and other addresses where the individual could receive mail, venues or agencies frequented or where the individual slept (if homeless), and a physical description completed by the interviewer. MSM who completed the survey received a $20 food voucher for the baseline assessment. We received human subjects' approval from the University of California, San Francisco Committee on Human Research. Written informed consent was obtained from all participants prior to administering the survey and locator form.

### Data Collection and Analysis

Project staff recorded perceived age range; race/ethnicity; gender; the types of services and products used by all participants by location and date of delivery, and repeat visits on each person who approached the van for services. Data were summarized in tabular form and frequencies were generated using the spreadsheet function of Microsoft Excel for windows. For the survey component, trained interviewers administered an anonymous questionnaire containing both open and closed ended items that captured socio-demographic data; self-reported HIV and sexually transmitted infection (STI) testing history and status, and sexual risk behaviors within the past 3 months. The survey also ascertained the number of sexual partnerships (i.e., the number of times a respondent engaged in risky "top" or "bottom" behavior with HIV positive or unknown status partners).

Injection and non-injection substance use were considered "ever used" and "used in the past 3 months." Frequency of injection drug use included categories from once a month to several times a day. Methamphetamine was defined as "meth, speed, ice, crank, or crystal."

Current or past participation in health or social service programs, including use of needle exchange programs, was assessed over the past 3 months. Recall periods were consistent with current studies conducted by the SFDPH to enable comparisons between similar populations on several measures. Additional measures were derived from an ongoing survey conducted by the SFDPH [35]. The survey was piloted with 4 MSM prior to fielding. Descriptive statistics and frequencies of key variables were generated using Statistical Analysis Systems software version 8 for windows (SAS Institute Inc, Cary, NC).

## Results

### Feasibility and process evaluation – general late night population

In 4 months, the LNBB engaged in condom distribution and resource referrals with over 600 individuals (duplicated count). Males accounted for 90% (58 unduplicated) of the outreach encounters in the South of Market site; 69% (207 unduplicated) in the Polk site, and 92% (140 unduplicated) in the Castro site. Repeat visits were made to each site: South of Market; 13%, Polk; 24% and Castro 17%. On average, 7 clients were seen each night over the course of the LNBB outreach.

Forty cases of water and juice and 25 cases of nutritional snacks were distributed; 4500 condoms and lubricants were dispensed. Approximately 2000 needles were exchanged and 200 packages containing 3 sterile syringes were provided to individuals who had no syringes to exchange. This procedure was followed by the LNBB to ensure consistency among all the needle exchange sites in San Francisco since these 3-syringe "starter packs" were permitted from all authorized needle exchange sites in San Francisco. The LNBB collected and safely disposed of approximately 1300 used syringes.

In the South of Market, needle exchange clients were 98% male; observed ethnicity was: 44% African American, 43% White and 9% Latino. In the Polk site, 90% of exchangers were male and observed as predominantly White (85%). In the Castro site, 94% of the exchangers were male and 78% were observed as White, 11% African American and 6% Latino. We engaged in as few as 2 and as many as 13 exchanges in a 4-hour period each night at each site.

Twenty-eight individuals expressed interest in HIV or STI testing as noted on outreach logs; however 7 declined citing a desire for anonymous or rapid testing for HIV and/or a desire for field based test results. Twenty-one individuals, 2 females and 19 males, were tested for HIV using Orasure.™ Two males tested positive for HIV antibodies. One male, newly identified as HIV positive, returned for his post-test counseling and results visit. Appropriate referrals to health care and social services were made. The second individual self-reported as HIV positive at the time of specimen collection; he did not return for his post-test counseling and results visit. Of the remaining 19 participants, 6 (29%) returned for HIV test results disclosure and post test counseling. Twelve males provided urine specimens for gonorrhea and Chlamydia testing; 4 returned for results. Results on all 12 STI tests were negative.

### Survey Results – MSM only

We intended to enroll 100 MSM for the pilot study. In a 4 month period, we screened 103 males; 73 self-reported having sex with men in the last 3 months and were therefore eligible for study participation; 55 were enrolled and 19 declined to participate primarily due to time limitations or their uncertainty of being able to follow up in 3 months. Of the 19 who declined, 63% were White; 21% African American; 10% Latino, and 5% Asian/Pacific Islander. Median age of the decliners was 35, just slighter older than the study population. There were no statistical differences on any of the screening variables between the men who declined and the men who were ultimately enrolled. Table 1 portrays the socio-demographic characteristics of the baseline study population.

**Table 1:** Socio-demographic characteristics of LNBB MSM in San Francisco

	N = 55 (unless noted)	%
Age (in years)		
18–25	16	29
26–35	12	22
36+	27	49
		
Ethnicity		
White	28	51
African American	8	15
Latino	13	24
Native American	4	7
Asian	1	2
Other	1	2
		
Sexual orientation		
Heterosexual	2	4
Homosexual	34	62
Bisexual	13	24
Other	6	11
		
Self reported HIV status		
Positive	16	29
Negative	33	60
Don't Know	6	11
		
Sources of income (figures exceed 100 % as subjects selected more than one source of income)		
Job	23	42
Govt. Benefits	28	51
Spouse, friend, family	13	24
Sex work	25	45
Scams/stealing/dealing	27	49
Street based economies (e.g. selling clothes, spare changing)	9	16
		
Education		
Less than high school	17	31
HS, GED, Tech, Voc	15	27
Some College	8	15
College	15	27
		
Current health insurance		
No	31	56
SF resident	47	85
Non- SF Resident	8	15
		
Living situation		
Stable*	16	29
Semi-Stable**	15	27
Unstable ***	24	44

* *Stable defined as "owning own home or paying rent for an apartment"*

** *Semi-stable defined as "living with someone and not paying rent, living in a hotel"*

*** *Unstable defined as "homeless"*

The survey sample was characterized by low levels of income and education, whose ages ranged from 18 – 55; median age was 32. Just under half (48%) of the sample were men of color. Over two-thirds (68%) of study participants fell into the lower-level income categories (i.e., between $0 and $1500/month). Almost two-thirds (62%) had lived in San Francisco for 5 years or more.

#### Substance Use

In terms of non injection drug use, 78% (n = 43) used MA and 69% (n = 38) used alcohol in the last 3 months. When asked about frequency of any MA use in the last 3 months, almost one-quarter of the participants reported using MA every day. Sixty-five percent (n = 36) of the participants reported a history of ever injecting drugs and 56% (n = 31) reported injecting drugs in the past 3 months. Of this latter group, all but one (97%) reported injecting MA. When asked about the frequency of injecting MA, 13% reported injecting several times a day in the last 3 months (Table 2).

**Table 2:** Sexual risk behaviors and drug use among LNBB MSM in San Francisco

	N	%
Partners past 3 months (n = 55)		
0	11	20
1–2	19	34
3 or more	25	46
		
Sexual behavior (n = 44)		
Only female partners	5	11
Only male partners	35	80
Both male and female partners	4	9
		
Non-injection drug use past 3 months (n = 55)		
		
Speed (methamphetamine, crank, crystal, ice)	43	78
Alcohol	38	69
GHB/Ketamine	23	42
Poppers	22	40
Crack	15	27
Viagra	14	25
Heroin	13	24
Ecstasy	10	18
Cocaine	9	16
Barbiturates	7	13
LSD	6	11
Other*	22	40
		
Injection drug use		
Ever	36	65
Past 3 months	31	56
		
Drugs injected past 3 months (n = 31)		
Heroin	11	35
Cocaine	6	19
Speed (methamphetamine, crank, crystal, ice)	30	97
Speedball (heroin & cocaine)	5	16
Goofball (speed & heroin)	4	13
Other**	5	16
		
Needle sharing "ever" (n = 36) (i.e., receptive sharing)	21	58
		
Needle sharing last 3 months (n = 31)***	11	35
		
Drug treatment (n = 55)		
Ever	32	58

*Other non- injection includes opiates, PCP, nitrous oxide

**Other injection includes crack, morphine

***The question was not worded to determine receptive or distributive sharing

Use of MA among participants varied across demographic categories and risk behaviors. Eighty-five percent of White participants reported MA use in the past 3 months whereas Latino and African American participants reported lower percentage of MA use at 69% and 57% respectively. All age groups were observed to have high levels of MA use but no statistical difference was found between the age groups. Participants between 26–35 years had the highest prevalence of MA use at 93%, followed by participants older than 35 years at 76%, and then participants 25 years and younger at 69%.

Non residents of San Francisco were much less likely to have used MA in the past 3 months (13%) than participants who resided in San Francisco (89%; (p < .001). A significant difference in MA use was also observed among homeless participants in which 91% of the group reported MA use compared with 69% of participants who were more stably housed (p < .05). Participants who reported participating in street economies (e.g., drug dealing, spare changing, stealing) were more likely to have used MA in the past 3 months (89%) than those who did not (60%; p < .01). Lack of health insurance was another socio-economic factor associated with MA; 90% of study participants who reported no health insurance used MA compared with 63% of insured participants (p < .05).

Of the 36 MSM who reported ever injecting, 75% reported using a needle exchange service. Other sources of accessing syringes, such as secondary exchange or from their friends, were also noted. All of the reported injectors (i.e., those who ever used, or used in the last 3 months) used needle exchange services from the van during LNBB outreach. The highest percentage (32%) of repeat needle exchanges occurred in the Castro neighborhood.

#### Sexual Behaviors and STIs

Almost half (46%) of the sample reported having three or more sexual partners during the last 3 months. Nineteen percent reported having an STI (e.g., syphilis, gonorrhea, Chlamydia, herpes, NGU, hepatitis B) in the previous 12 months; 20% reported having hepatitis C (HCV) and 47% had been tested for HCV in the past 12 months. Sixty-four percent had been vaccinated for hepatitis B and hepatitis A (HBV/HBA). All participants had been tested for HIV.

#### Sexual Behaviors and Substance Use

Participants were asked to report on sexual activity with up to five of their recent sex partners and their use of substances during sex. Of the 29 unprotected receptive anal sexual encounters reported by 11 HIV negative participants, 20 (69%) of the encounters were with an HIV positive or unknown status partner. Of the 25 unprotected receptive anal sexual encounters while high on alcohol or drugs, 15 (60%) were with an HIV positive or unknown status partner.

Among the 13 self-reported HIV positive participants, potential HIV infection from insertive anal intercourse to an HIV negative or unknown status partner was also reported. Ten of the 13 HIV positive participants reported insertive anal intercourse, totaling 39 encounters. Thirty-five encounters (90%) were unprotected of which 14 (36%) were with an HIV negative or unknown HIV status partner. Eleven of the 14 unprotected insertive encounters were with an HIV negative or unknown status partner while the respondent was high. Alcohol and MA were the most commonly reported substances used by both HIV positive and HIV negative MSM during sexual activity.

## Discussion

The LNBB corroborated earlier findings of a larger sero-prevalence study among a similar population, and established an effective methodology for reaching a high risk population of MA-using MSM, half of whom were injection drug users (IDUs). We believe an extended field presence (i.e., longer than 4 months) is needed to establish credibility, particularly among MSM-IDUs precisely because the majority of study participants were recruited in the last 6 weeks of the project. Longer field time could have produced higher levels of study participation and higher follow up rates for HIV/STI test results. We were able to follow up with 31 (56%) of our study participants largely due to a project coordinator with previous experience serving similar populations.

The LNBB reached a subpopulation of MSM with documented high risks for HIV, HCV and other STIs through injection drug use and sexual behavior. Unprotected anal intercourse with an HIV discordant partner is an important risk factor for HIV transmission; the level of unprotected anal intercourse was high among all LNBB participants. Furthermore, sexual positioning analysis by HIV status revealed that the potential of transmission from an HIV positive individual to an uninfected partner was also high. Nearly 70% of all the episodes of unprotected receptive anal intercourse by HIV negative participants were with a "top" partner whose HIV status was positive or unknown. Conversely, 36% of all the unprotected insertive anal sexual encounters reported by HIV positive participants were with a "bottom" partner whose HIV status was HIV negative or unknown. We included partners whose HIV status was unknown in these risk analyses largely to address the explicit messages in current risk reduction interventions that advocate knowledge of partner HIV status when negotiating safe sex practices. Clearly, significant numbers of MSM in this population were not using condoms when engaging in anal intercourse. Further research should focus on understanding the relationship between high risk HIV discordant sexual intercourse and variables associated with MA and/or polydrug use.

### Limitations

The chief limitation of the LNBB lies in convenience sampling and a baseline population of 55 MSM. Nineteen self-reported MSM declined to participate; and this could have established selection bias in the study sample. Eleven men were screened into the study as eligible participants; however during data cleaning, we discovered that they reported no sexual partners or only female partners in the last 3 months. These 11 men were excluded from the sexual behavior analysis; however we chose to include them in all other analyses of substance use and service utilization. Few study participants or service clients accessed specific harm reduction counseling services beyond needle exchange, although interviewers frequently provided harm reduction advice and techniques during survey administration. Rapid testing for HIV was not yet available during the study period; therefore the low uptake and return rate for HIV/STI could be due to our reluctance to provide test results and post test counseling in a field based setting. We believe these limitations do not negate the policy and practice implications of the LNBB.

We found no comparable studies of late night outreach to MSM; however the meta-analyses related to outreach among the homeless and injection drug users are relevant to the methodology employed in this study [41]. The LNBB provided the first legally sanctioned late night needle exchange service in San Francisco. We are aware of this type of service in Canada and Australia [42-44], but are unaware of late night services elsewhere, particularly in California. Other studies of roving and van based needle exchange have highlighted the need for varied methods of outreach and service provision to attract different subpopulations of injection drug users and to establish needle exchange sites beyond fixed sites. In these studies, populations reached were distinguished as having more frequent injection patterns; fewer years of injecting; more difficulty in accessing clean needles, and in general reporting high risk behaviors [45-47]. Our population of MSM was similar to these populations in terms of injection drug use and high risk sexual behaviors.

## Conclusion

The LNBB demonstrated the feasibility, acceptability and cost efficiency of a local health department providing late night harm reduction services to a disenfranchised high risk population of MSM. On a limited budget (e.g., within US$100,000) in a condensed timeframe, we established what we believe is the obligation of a local health jurisdiction to provide late night needle exchange for MSM and other IDU where this service is legally sanctioned.

The three staff discussed in this study were required to work every Friday, Saturday and Sunday from midnight (i.e., to set up and stock the van) through 6 a.m. (i.e., to restock and store the van) over a 5-month period (one month pilot and 4 months of study implementation). We recommend that future studies or late night harm reduction interventions use volunteers or rotate a larger pool of staff to diminish the burden on a small cadre of outreach staff.

Recent trends in the HIV/AIDS epidemic in San Francisco, related studies and programmatic experience have resulted in discussions among policy makers, HIV prevention and drug treatment providers regarding the potential replication of late night, mobile harm reduction for MSM and other IDUs in San Francisco.

## Competing interests

The author(s) declare that they have no competing interests.

## Authors' contributions

VR, HFR and TAK drafted the manuscript. TK led data analysis. WMcF reviewed and approved the final version.
